# Salinomycin suppresses TGF-β1-induced EMT by down-regulating MMP-2 and MMP-9 via the AMPK/SIRT1 pathway in non-small cell lung cancer

**DOI:** 10.7150/ijms.50080

**Published:** 2021-01-01

**Authors:** Ki-Eun Hwang, Hyo-Jin Kim, In-Sol Song, Chul Park, Jae Wan Jung, Do-Sim Park, Seon-Hee Oh, Young-Suk Kim, Hak-Ryul Kim

**Affiliations:** 1 Department of Internal Medicine, Wonkwang University, School of Medicine, Iksan, Jeonbuk 54538, Republic of Korea; 2 Department of Laboratory Medicine, Wonkwang University, School of Medicine, Iksan, Jeonbuk 54538, Republic of Korea; 3 Department of Premedicine, Chosun University, School of Medicine, Gwangju 61452, Republic of Korea; 4 Medical Convergence Research Center, Wonkwang University, Iksan, Jeonbuk 54538, Republic of Korea

**Keywords:** Salinomycin, TGF-β1, EMT, AMPK, SIRT, MMP, Lung cancer

## Abstract

Salinomycin (Sal) is a recently identified anti-tumor drug for treating several types of solid tumor; however, its effects on the migratory and invasive properties of non-small cell lung cancer (NSCLC) remain unclear. This study investigated the inhibitory effect underlying mechanisms of Salon transforming growth factor-β1 (TGF-β1)-induced epithelial-to-mesenchymal transition (EMT) and cell migration. Sal solidly blocked cell migration and invasion enhancement by TGF-β1-induced EMT, through recovering E-cadherin loss and suppressing mesenchymal markers induction, as well as TGF-β1-mediated AMPK/SIRT signaling activity upregulation. The pharmacologic inhibition or knockdown of AMPK or SIRT1 can act synergistically with Sal to inhibit TGF-β1-induced MMP-2 and MMP-9. In contrast, AMPK or SIRT1 upregulation can protect against TGF-β1-induced MMP-2 and MMP-9 inhibition by Sal. Next we demonstrated that the MMP-2 and MMP-9 knockdown can act synergistically with Sal to inhibit TGF-β1-induced EMT. Moreover, treatment of PMA of MMP activator increased TGF-β1-induced MMP-2 and MMP-9, even with Sal. Our results demonstrate that Sal suppresses TGF-β1-induced EMT by downregulating MMP-2 and MMP-9 through the AMPK/SIRT pathway, thereby inhibiting lung cancer cell migration and invasion.

## Introduction

Non-small cell lung cancer (NSCLC) accounts for approximately 80% of all lung cancers, and few patients achieve long-term survival despite treatment advances over the past few decades. Metastasis, invasion, and drug resistance are the main factors contributing to relapse and death 1.

Epithelial-to-mesenchymal transition (EMT) is a process where cells undergo morphologic changes from a polarized epithelial phenotype to a highly motile mesenchymal phenotype. The switch of E-cadherin to N-cadherin is the critical event in EMT that makes single cells more motile and invasive 2-4. Cells undergoing EMT also increase mesenchymal proteins synthesis and matrix metalloproteases (MMPs) expression. These extracellular matrix components stimulate integrin signaling and facilitate cell migration 5, 6. Therefore, elucidating the molecular mechanism that regulates E-cadherin, N-cadherin, and MMP expression has become pivotal for understanding cancer invasion and metastasis.

Transforming growth factor-β1 (TGF-β1) is involved in many biological processes, such as embryogenesis, wound healing, cell proliferation, differentiation, and EMT 7-9. In cancer, TGF-β acts as a suppressor in the early stages of tumorigenesis by inhibiting cell growth and inducing cell apoptosis. Conversely, in the later stages of tumor progression, it acts as a promoter, as TGF-β suppresses the tumor cells' ability to be growth-arrested while undergoing EMT, which correlates with the increased invasiveness and metastasis 10.

Salinomycin (Sal) has been globally used in animal husbandry for many years. It is a potassium ionophore, isolated from *Streptomyces albus*, and was initially considered an antibiotic 11. Recent studies show that Sal regulates apoptosis, proliferation, and differentiation in several cell types 11, 12. A novel method has been developed for identifying agents that targeted breast cancer stem cells (CSCs) from approximately 16,000 compounds Gupta et al. 13. Also Sal reportedly kills breast CSCs more effectively than the conventional anti-tumor drug paclitaxel. Sal's ability to eradicate CSCs, modulate EMT, and suppress the invasion capacity of several types of tumors has been reported in recent years 14-16. However, the effects of Sal on the migratory and invasive properties of NSCLC cells and its underlying, remain unclear.

In the present study, we showed that MMP-2 and MMP-9 activity mediates Sal's anti-invasive and anti-migratory effects by downregulation through the AMPK/SIRT pathway, thereby inhibiting the invasion and metastasis of lung cancer cells.

## Materials and Methods

*Materials.* Roswell Park Memorial Institute medium 1640 (RPMI 1640), fetal bovine serum (FBS), and antibiotics (penicillin and streptomycin) were obtained from GIBCO BRL Co. (Grand Island, NY, USA). Salinomycin, 3-(4,5-dimethyl-2-thiazolyl)-2,5-diphenyl-2H-tetrazolium bromide (MTT), propidium iodide (PI), dimethyl sulfoxide, Sirtinol, and AICAR were purchased from Sigma (St. Louis, MO, USA). Recombinant human TGF-β1 was purchased from R&D Systems (Abingdon, UK). Affinity-purified monoclonal antibodies against mouse SIRT1 antibody were obtained from Abcam (Cambridge, UK). AMPK inhibitor Compound C was purchased from Calbiochem (San Diego, CA, USA). MMP total activator PMA was purchased from Merck Millipore (Billerica, MA, USA). Antibodies against E-cadherin, N-cadherin, MMP-2, MMP-9, vimentin, phospho-AMPK**,** and AMPK were purchased from Cell Signaling Technology (Beverly, MA, USA). Anti-rabbit IgG-conjugated horseradish peroxidase (HRP) antibodies and enhanced chemiluminescence (ECL) kits were purchased from Amersham Pharmacia Biotech (Buckinghamshire, UK).

*Cell culture and viability test.* Lung cancer cell lines A549 and H460 were purchased from the American Type Culture Collection (ATCC). These cell lines were grown in RPMI 1640 containing 100 units/mL penicillin, 0.1 mg/mL streptomycin, and 10% FBS. Cells were incubated in a humidified atmosphere of 5% CO_2_ in air at 37 °C and maintained in log growth phase. Cell viability was determined by the MTT assay. After the cells were treated with the specified drugs, MTT was added to the cell suspension and incubated for 4 h. The cells were then washed three times with phosphate-buffered saline (PBS; pH 7.4), and the insoluble formazan product was dissolved in dimethyl sulfoxide. The optical density (OD) at 595 nm was measured for each well using a microplate reader (Titertek Multiskan; Flow Laboratories, North Ryde, New South Wales, Australia). The OD resulting from formazan production in control cells was taken to denote 100% cell viability, and all other measurements were expressed as a percentage of this control value.

*Tumor xenograft studies in nude mice.* Five- to six-week-old BALB/c athymic nude mice (Charles River, Japan) were housed in cages with HEPA-filtered air (12-h light/dark cycle) and had *ad libitum* access to food and autoclaved water. H460 or H460 cells plus TGF-β1 was injected subcutaneously (*s.c.*) into both hind legs of each mouse. The mice were randomly assigned to three experimental groups (n = 5 each) when the implanted tumors reached a volume of 90-130 mm^3^. Each group was monitored until tumors reached a volume of 1,300 mm^3^, or for 21 d.

*Scratch-migration assay.* A549 and H460 cells were cultured in 6-well dishes (seeding density 1 × 10^6^ cells/well). Confluent cell monolayers were disrupted by standardized wound scratching using a sterile 200-μL pipette tip and incubated in a culture medium with 1% FBS, with or without 5 ng/mL TGF-β1, 10 μM Sal, TGF-β1 plus 5 μM Sal, or TGF-β1 plus 10 μM Sal, for 48 h. Migration of cells into the bare area and recovery of the monolayer was evaluated every 12 h for 48 h using a phase contrast microscope, and was digitally photographed (Nikon Diaphot 300; Nikon, Tokyo, Japan).

*Electric cell-substrate impedance sensing (ECIS) wound healing assay.* Wound healing assays were performed using ECIS (Applied BioPhysics, Troy, NY, USA) technology, following our previously established protocol 17. For wound healing assays, confluent A549 cells monolayers cultured on ECIS plates were subjected to an elevated voltage pulse of 60 kHz frequency, 3.5 V amplitude, and 30 s duration, leading to the death and detachment of cells present on the small active electrode, resulting in a wound normally healed by cells surrounding the small active electrode that have not been subjected to the elevated voltage pulse. Wound healing was then assessed by continuous resistance measurements for 24 h.

*Matrigel invasion assay.* Cell invasion assay kits (Chemicon International, Temecula, CA) were used to detect cell invasion, following the manufacturer's protocol. Cells were resuspended in culture media and incubated in a chemo invasion chamber. A549 and H460 cells were seeded at a density of 2 × 10^4^ per insert and cultured for 12 h. Next, the cells were placed in wells containing the same medium plus TGF-β1 (5 ng/mL), with or without Sal. After 48 h, non-invading cells were removed using cotton swabs. The invasive capability of cells was measured as per the manufacturer's recommendations. Photomicrographs of the invasive cells were taken in five predetermined fields (magnification 200×) and quantification of the stained cells was performed by dissolving cells in 10% acetic acid and measuring the OD at 540 nm.

*ECIS invasion assay.* Electrode arrays were obtained from Applied BioPhysics (Troy, NY, USA), and ECIS invasion assays were performed as described previously 18. ECIS array wells were precoated with a solution of 200 µg/mL gelatin in 0.15 mol/L NaCl. After 15 min of incubation to allow for gelatin adsorption, the gelatin solution was aspirated, and the electrode-containing wells were rinsed twice with PBS. They were then partially filled with 200 µL of human umbilical vein endothelial cell (HUVEC) medium and allowed to equilibrate for 15 to 60 min in a humidified CO_2_ incubator. Approximately 1 × 10^5^ HUVECs were added to each well in 200 µL HUVEC medium. The attachment and spreading of cells into the ECIS wells was followed by impedance measurements using ECIS. HUVECs were challenged with monodispersed cell suspensions of A549 cells (20 × 10^5^/mL) in 50 µL of fresh HUVEC medium. Triplicate wells were used for each treatment. The cells were treated with 1% FBS, with or without TGF-β1, Sal, and TGF-β1 plus Sal. The impedance of the challenged endothelial cell layer was monitored via ECIS for the next 12-40 h.

*Western blotting.* Cells were harvested and lysed in radioimmunoprecipitation assay buffer (50 mM Tris-Cl [pH, 7.4], 1% NP40, 150 mM NaCl, 1 mM EDTA, 1 mM phenylmethylsulfonyl fluoride, 1 µg/mL each of aprotinin and leupeptin, and 1 mM Na_3_VO_4_). After centrifugation at 12,000 ×*g* for 30 min, the supernatant was collected, and the protein concentration was determined by the Bradford method (Bio-Rad Protein Assay; Hercules, CA, USA). Equal amounts of protein were separated by 10% sodium dodecyl sulfate-polyacrylamide gel electrophoresis (SDS-PAGE) under reducing conditions and were subsequently transferred to polyvinylidene difluoride membranes. The membranes were blocked with 5% skim milk in TBS-T (25 mM Tris [pH, 7.6], 138 mM NaCl, and 0.05% Tween-20) for 1 h and probed with primary antibodies (at 1:1000-1:5000). After washing, the membranes were incubated with the relevant HRP-conjugated secondary antibody (at 1:2000-1:10,000). Immunoreactive signals were detected using an ECL detection system.

*Gelatin zymography*. To analyze the activity of MMP-2 and MMP-9, we respectively incubated A549 and H460 cells (1 × 10^5^ cells/well) in a 24-well plate for 24 h. After serum starvation for 24 h, the supernatant was collected after treatment with TGF-β1 in the absence or presence of Sal, and subjected to SDS-PAGE in 10% polyacrylamide gel with 1 mg/mL gelatin. After electrophoresis, the gels were incubated in 2.5% Triton X-100 (1 h, 37 °C), followed by overnight incubation in 50 mM Tris-HCl (pH 7.8), 5 mM CaCl_2_, 0.02% NaN_3_, and 0.02% Brij gels. They were stained with 2.5% Coomassie Blue R-250 (Bio-Rad) for 45 min, followed by destaining in deionized water with 10% acetic acid and 20% methanol. The gels were scanned and density analysis of the bands was performed using Photoshop CS4.0 (Alphalmager 2000, Alpha Innotech, San Leandro, CA).

*Gene silencing.* Pooled small interfering RNA (siRNA) oligonucleotides against SIRT1 were purchased from Cell Signaling Technology, and AMPK, MMP-2, and MMP-9 were purchased from Life Technologies. Twenty-four hours after seeding, the cells were transfected with 100 nM pooled oligonucleotide mixture using Lipofectamine 2000 (Invitrogen), according to the manufacturer's protocol. Twenty-four hours after transfection, the media were removed, and cells were treated with the indicated drugs. Gene silencing efficacy by siRNA was assessed by western blot analysis.

*Preparation of the recombinant adenovirus.* To prepare SIRT1-expressing adenovirus, human SIRT1 cDNA was cloned into the KpnI and XhoI sites of pENTR 2B (Invitrogen), and the SIRT1 cDNA insert was transferred to the pAd/CMV/V5-DEST vector by the Gateway system using LR Clonase (Invitrogen). The plasmids were linearized with PacI (Promega, Madison, WI) and transfected into A549 and H460 cells using Lipofectamine 2000 (Invitrogen). As a control, the pAd/CMV/V5-GW/lacZ vector (Invitrogen) was used to produce lacZ-bearing adenovirus.

*Statistical analysis.* Each experiment was performed at least thrice, and all the values were expressed as the mean ± standard deviation (SD) of triplicate samples. Student's *t*-test was used to determine statistical significance. Values with *p* < 0.05 were considered statistically significant.

## Results

**Figure 1:**
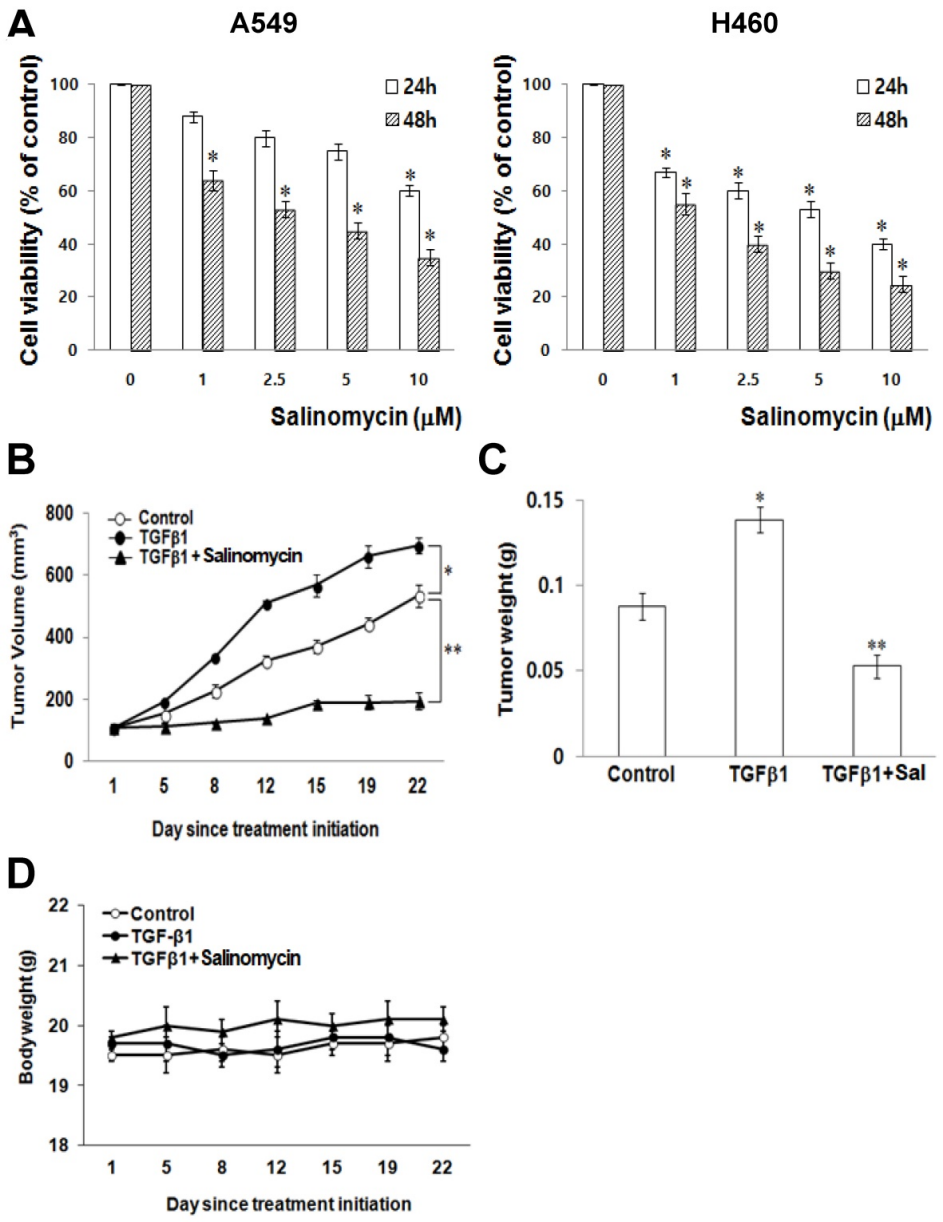
Sal's effects on NSCLC cells growth. (A) A549 and H460 cells were treated with different concentrations of Sal for 24 or 48 h, and viability was determined using the MTT assay. The viability of control cells was set at 100%, and survival relative to the control is presented. The data represent the means ± S.D. of three independent experiments. ^*^, *p* < 0.05, compared with the control. (B) Athymic nude mice were subcutaneously injected with 2 × 10^6^ H460 cells (0.2 mL cell suspension) in both hind legs. When the implanted tumors reached a volume of 90-130 mm^3^, mice were assigned randomly to one of the following experimental groups (n = 5 each): no treatment, TGF-β1, and TGF-β1 plus Sal (5 mg/kg, intraperitoneally, daily for three weeks). Tumor dimensions were measured three times a week. The tumor volume was estimated using the formula: volume = L × W^2^/2. Points, mean of five animals; bars, SD. ^*^, *p* < 0.01, compared with the control.(C)The tumors were weighed. The data represent the mean ± SD of five animals. ^*^, *p* < 0.01, compared with the control. (D) Body weights of the mice were insignificantly different among all groups.

*The Effects of Sal on the cytotoxicity of NSCLC cells.* To test Sal's effects on NSCLC cell viability, we treated the lung cancer cell lines, A549 and H460, with a range of Sal doses (0, 1, 2.5, 5, and 10 μM) for 24 h and 48 h. We observed that Sal had asignificanteffect, inhibiting A549 and H460 growth (Figure 1A) in a dose- and time-dependent manner.

*Sal suppresses lung cancer tumor growth in vivo.* To demonstrate Sal's inhibitory effects on tumor growth in xenografts, we performed an anti-tumor study using athymic nude mice subcutaneously injected with H460 cells plus TGF-β1. The animals in the experimental group were intraperitoneally injected with Sal (5.0 mg/kg) every day, whereas those in the control group were injected with PBS.

Sal treatment significantly reduced tumor growth compared with the control group (Figure 1B). Furthermore, the mice injected with Sal had a considerably smaller tumor volume than the controls (Figure 1C). Mice body weight on subsequent days after the first drug injection was not significantly different among the experimental groups (Figure 1D).

**Figure 2:**
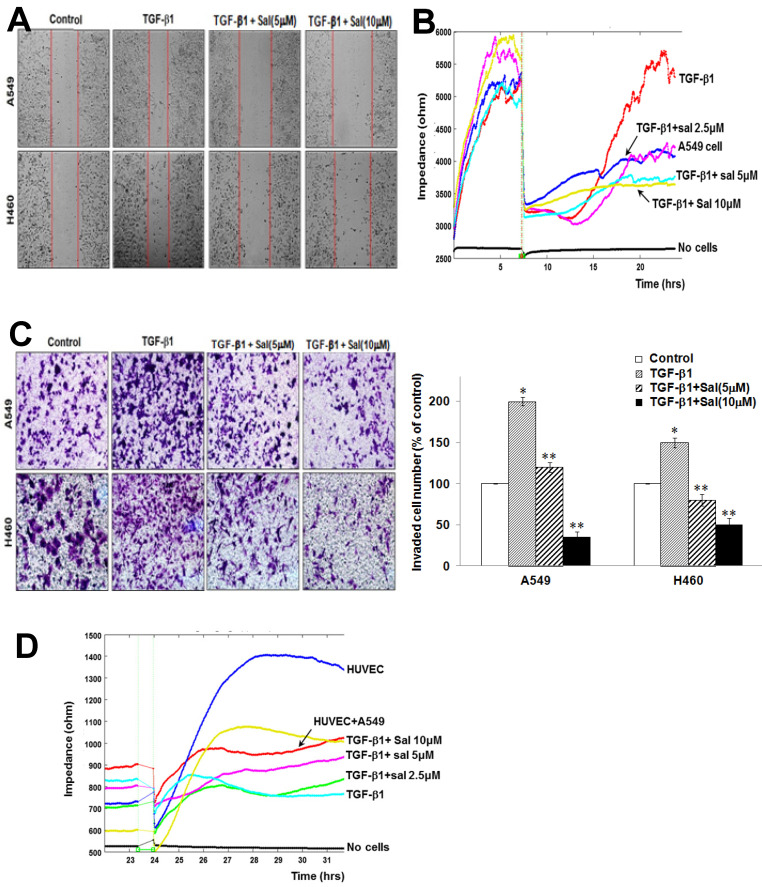
Sal's effects on TGF-β1-induced A549 and H460 cell migration and invasion. (A) Cell migration was evaluated by scratch assay. The confluent A549 and H460 monolayer was scratched with a pipette tip and washed to remove debris. Fresh medium containing 0.5% serum was then added. Red lines indicate cell edges at the T_0_ point. Representative pictures are shown. (B) For the ECIS migration assay, A549 cells were stimulated with 5 ng/mL TGF-β1 for 2 h and then incubated with 0, 2.5, 5, and 10 µM Sal for 48 h. Then, cell migration was assessed by continuous resistance measurements for 40 h. (C) Effects of Sal on A549 and H460 cell invasion in a 200× light microscope after crystal violet staining by Matrigel invasion assay, as described in Materials and Methods. Matrigel invasion of A549 and H460 cells, as counted in five random views. The data represent the mean ± SD of three independent experiments. ^*^, *p* < 0.01, compared with the control;^**^, *p* < 0.05, compared with the TGF-β1 group. (D) For the ECIS invasion assay, resistance changes in the impedance were recorded at 4 kHz, as confluent layers of HUVECs were challenged with A549 cell suspensions. The control curve represents HUVECs that received media without A549 cells. A549 cells were treated as described in Material and Methods, and resistance changes were monitored for 40 h.

*Sal inhibits TGF-β1-induced A549 and H460 cell migration.* We assessed Sal's effects on migration using scratch-migration assay and ECIS wound healing assay to investigate its potential role in inhibiting lung cancer cell migration. TGF-β1 enhanced A549 and H460 cell migration, whereas Sal dose-dependently inhibited it, as observed in a conventional scratch-migration assay (Figure 2A). Using an ECIS-based quantitative real-time assay to investigate A549 cell migration, we confirmed that TGF-β1-treated cells showed increased resistance, whereas the combined Sal plus TGF-β1 treatment showed decreased resistance (Figure 2B). These results indicate that Sal effectively prevention of TGF-β1-induced lung cancer cell migration.

*Sal inhibits TGF-β1-induced A549 and H460 cell invasion.* We investigated Sal's effects on lung cancer cell invasion after TGF-β1 stimulation using a Matrigel invasion assay and an ECIS invasion assay. TGF-β1 treatment increased A549 and H460 cell invasion, compared with untreated cells, whereas Sal treatment inhibited lung cancer cell invasion in a dose-dependent manner. The quantitative analysis further confirmed Sal's inhibitory effect on invasion (Figure 2C).

In the ECIS-based invasion assay, established human umbilical vein endothelial cell (HUVEC) layers were challenged using A549 cells. The decreased resistance indicated that direct interactions occurred between the A549 cells and the HUVECs, leading to A549 cell extravasation on the substratum. TGF-β1 treatment induced a steep drop in resistance compared with untreated controls, demonstrating that TGF-β1 increased invasiveness. Sal dose-dependently inhibited the invasive potential of A549 cells even with TGF-β1 (Figure 2D). These results indicated that Sal could effectively inhibit TGF-β1-induced lung cancer cell invasion.

**Figure 3:**
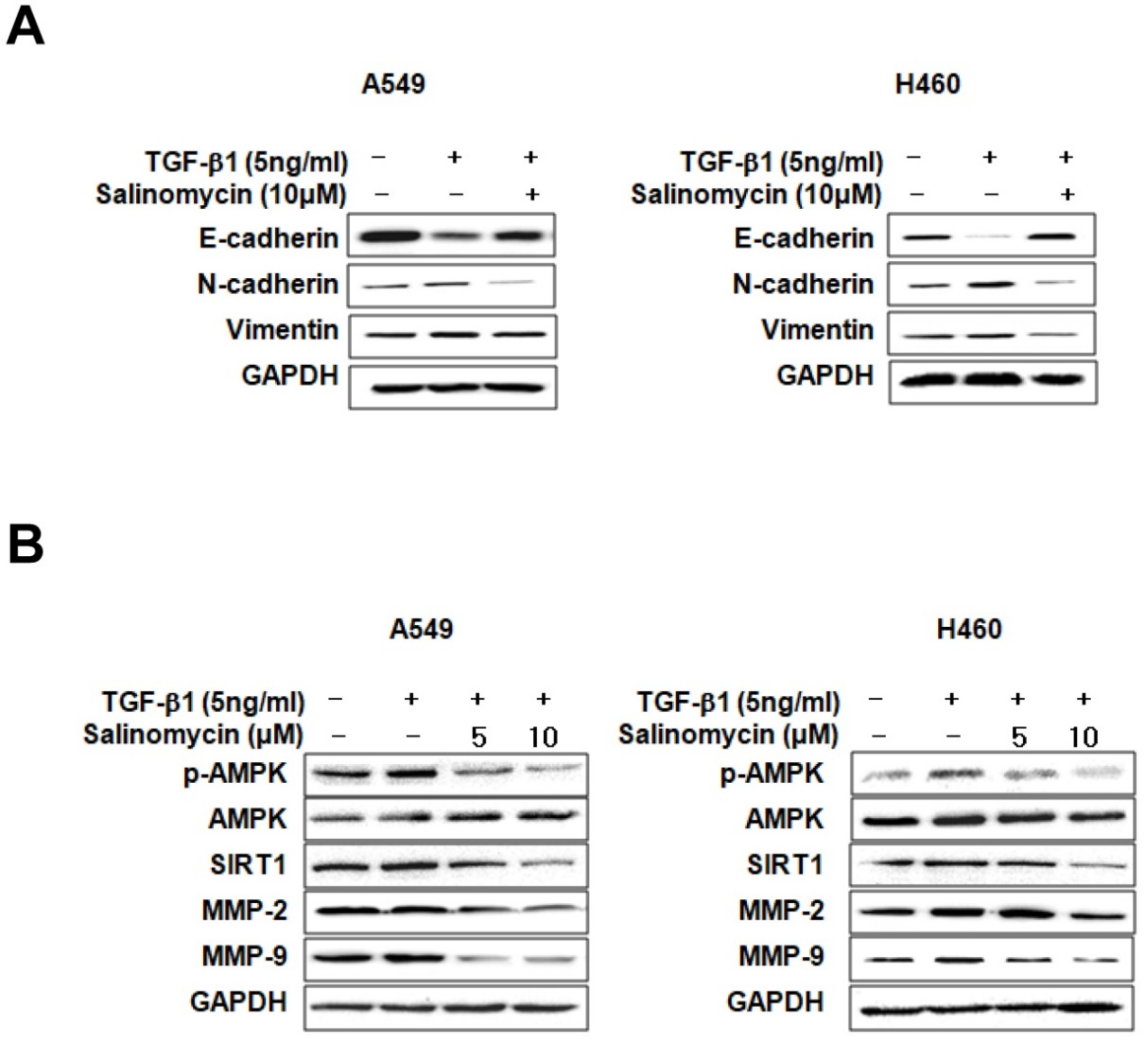
Effects of Sal on TGF-β1-induced EMT through the AMPK/SIRT1 signaling pathway. (A) A549 and H460 cells were treated with 5 ng/mL TGF-β1 and 10 µM Sal for 48 h. Cell lysates were used to measure E-cadherin, N-cadherin, and vimentin level. (B) The cells were treated with 5 ng/mL TGF-β1 and Sal (5 or 10 µM), and AMPK/SIRT1-related protein levels were examined through immunoblotting. Similar data were obtained from three independent experiments.

*Sal inhibits TGF-β1-induced EMT through the AMPK/SIRT1 signaling pathway.* We examined EMT marker expression in TGF-β1- or TGF-β1 plus Sal treated NSCLC cells to investigate Sal's effects on TGF-β1-induced EMT. E-cadherin expression declined in response to TGF-β1, whereas N-cadherin and vimentin expressions increased. However, Sal reversed vimentin-induced TGF-β1expression and inhibited the TGF-β1-induced cadherin switch (Figure 3A). Several studies have reported that the TGF-β1-induced SIRT1/AMPK signaling pathways are consistent with tumorigenicity 19, 20. Next, we examined the effects of Sal on TGF-β1-induced AMPK/SIRT1 activation in A549 and H460 cells. TGF-β1 induced SIRT1 expression levels and AMPK phosphorylation levels, as well as MMP-2 and MMP-9 expression levels, in NSCLC cells, In contrast, Sal treatment suppressed the TGF-β1-induced SIRT1 expression and AMPK phosphorylation in a concentration-dependent manner (Figure 3B).

**Figure 4:**
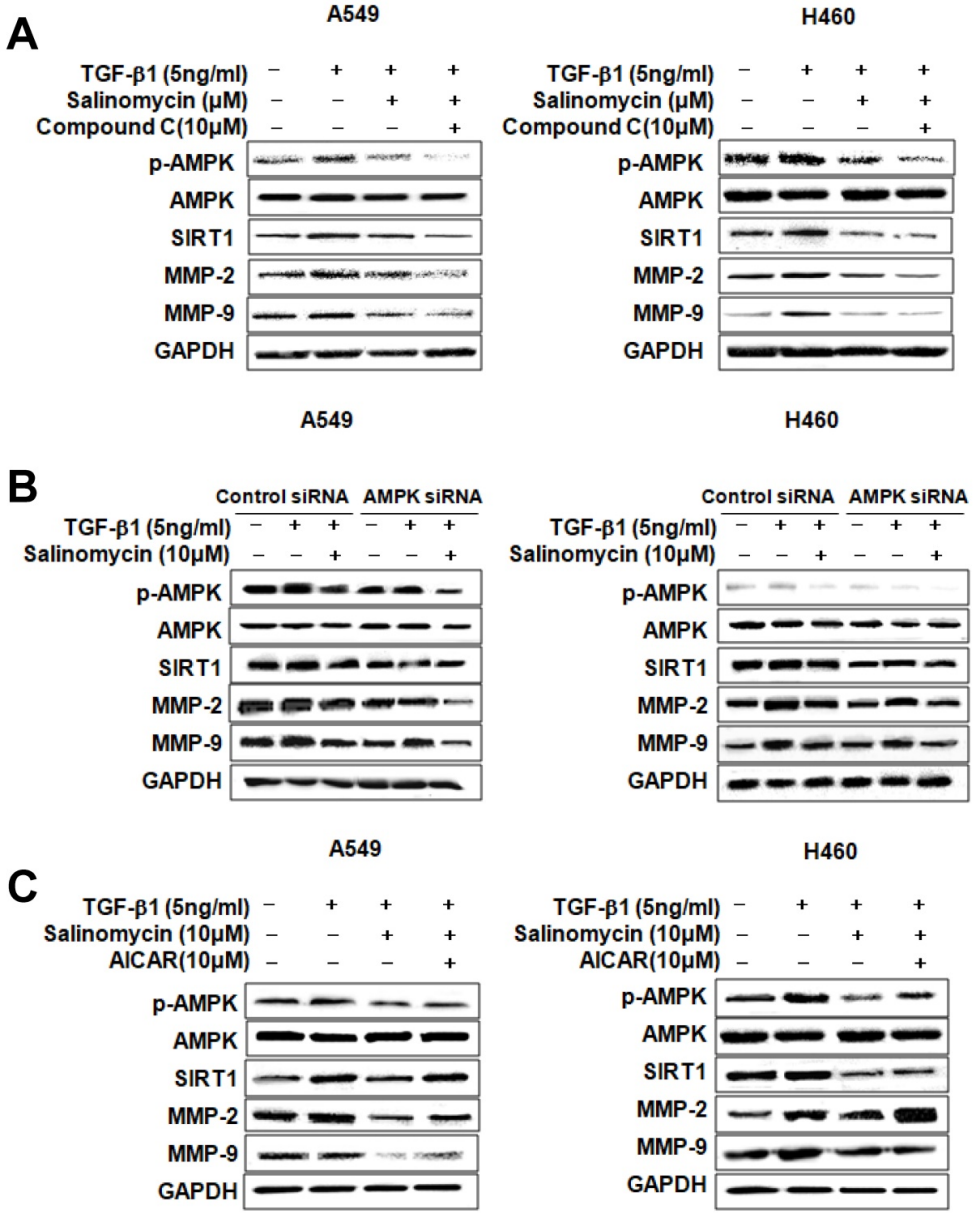
AMPK involvement in TGF-β1-induced AMPK/SIRT1 signaling by Sal. (A, B) The Effects of AMPK inhibition on TGF-β1-induced AMPK/SIRT1 signaling. A549 and H460 cells were treated with an AMPK inhibitor, Compound C, or transfected with AMPK siRNA, and then further incubated in the presence of Sal for 24 h. The cell lysates were routinely prepared, and changes in signaling-mediated hallmarks were determined through Western blotting. (C) The cells were treated with an AMPK activator, AICAR, and then further incubated in the presence of Sal for 24 h.

*Involvement of AMPK in the inhibition of TGF-β1-induced MMP-2 and MMP-9 expression by Sal.* To determine whether p-AMPK inhibition affected MMP-2 and MMP-9 expression, we treated the cells with Sal, followed by Compound C, an AMPK inhibitor. Compound C expectedly enhanced Sal's suppressive effect on TGF-β1-induced MMP-2 and MMP-9 expression in NSCLC cells, as revealed through Western blotting (Figure 4A). To rule out the chemical inhibitor's non-selective effects, we examined how Sal's TGF-β1-induced MMP-2 and MMP-9 downregulation affects AMPK siRNA-transfected cells. AMPK silencing, in combination with Sal treatment, augmented the downregulation of TGF-β1-induced MMP-2 and MMP-9 expression in A549 and H460 cells (Figure 4B). These findings reveal that pharmacologic inhibition or AMPK knockdown synergistically acted with Sal to inhibit TGF-β1-induced MMP-2 and MMP-9 expression.

Next, we examined whether AMPK upregulation could confer protection against Sal's inhibitory effect on TGF-β1-induced MMP-2 and MMP-9 expression. As shown in Figure 4C, the treatment of AICAR of AMPK activator increased TGF-β1-induced MMP-2 and MMP-9 expression, in NSCLC cells, even in the presence of Sal. These results indicate that AMPK is involved in inhibiting TGF-β1-induced MMP-2 and MMP-9 expression by Sal.

**Figure 5:**
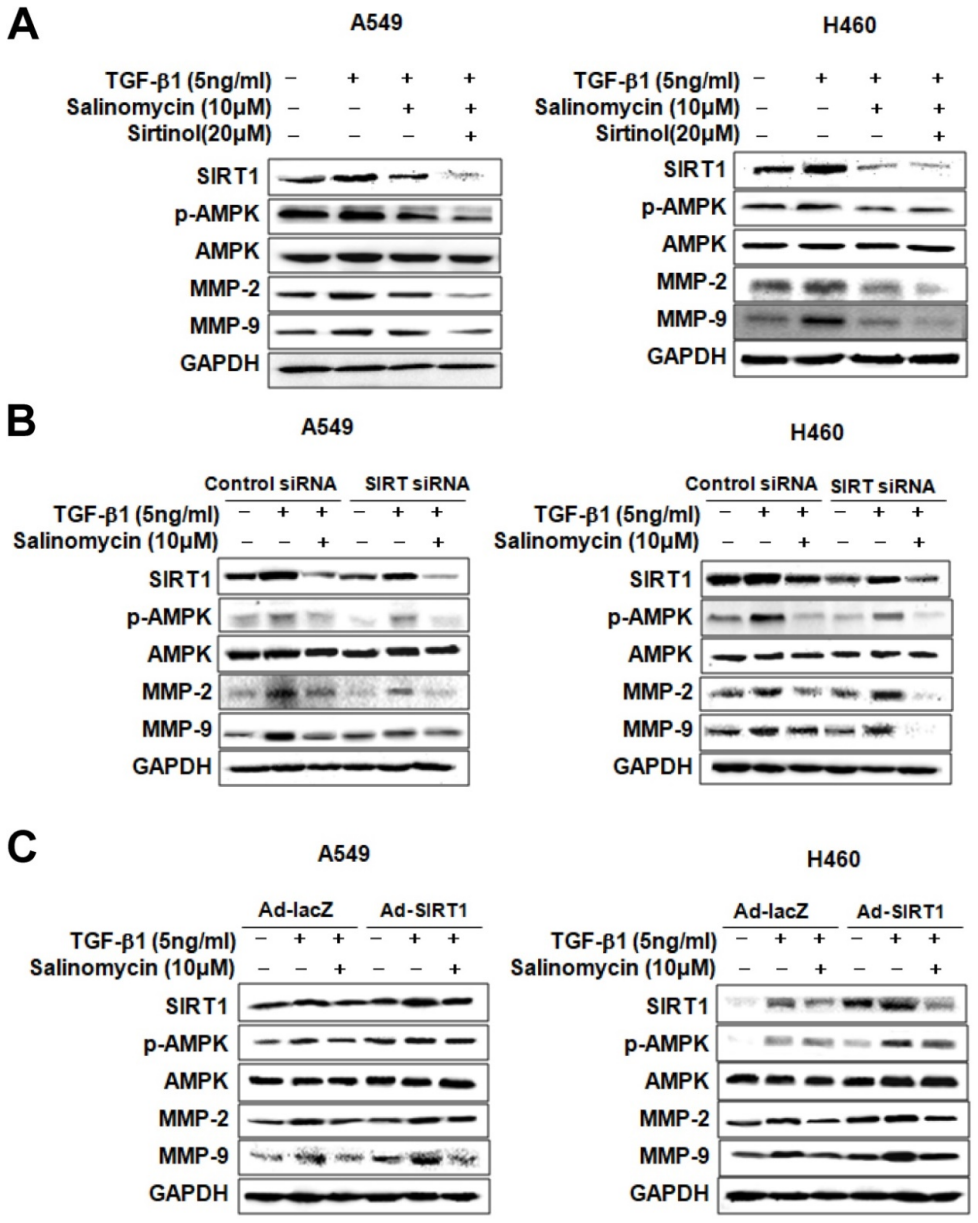
SIRT1 involvement in TGF-β1-induced AMPK/SIRT1 signaling by Sal. (A, B) Effects of SIRT1 inhibition on TGF-β1-induced AMPK/SIRT1 signaling. The cells were treated with a SIRT1 inhibitor, sirtinol, or transfected with SIRT1 siRNA, and then further incubated in the presence of Sal for 24 h. (C) Effects of SIRT1 activation on TGF-β1-induced AMPK/SIRT1 signaling. The cells were transfected with Ad-lacZ or Ad-SIRT1 and then further incubated in the presence of Sal for 24 h. The cell lysates were routinely prepared, and alterations in signaling-mediated hallmarks were determined through Western blotting.

*Involvement of SIRT1 in inhibiting TGF-β1-induced MMP-2 and MMP-9 expression by Sal.* We treated the cells with pharmacologic SIRT1 inhibitor, sirtinol, or knocked down SIRT1 by introducing as iRNA for SIRT1to determine the role of SIRT1 in inhibiting TGF-β1-induced MMP-2 and MMP-9 expression by Sal. When TGF-β1-induced A549 and H460 cells were treated with sirtinol plus Sal, MMP-2, and MMP-9 expression was attenuated (Figure 5A). In the same way, Figure 5B shows the attenuating effect of SIRT1 siRNA plus Sal treatment on TGF-β1-induced MMP-2 and MMP-9 expression. These findings indicated that the SIRT1 pharmacologic inhibition or knockdown could act synergistically with Sal to inhibit TGF-β1-induced MMP-2 and MMP-9 expression.

We also examined whether SIRT1 upregulation could block TGF-β1-induced MMP-2 and MMP-9 inhibition by Sal. For this, we introduced a SIRT1-expressing adenovirus into A549 and H460 cells. As shown in Figure 5C, SIRT1 overexpression treatment markedly increased TGF-β1-induced MMP-2 and MMP-9 expression in A549 and H460 cells, even in the presence of Sal. Altogether, these results indicate that SIRT1 is involved in inhibiting TGF-β1-induced MMP-2 and MMP-9 expression by Sal.

**Figure 6:**
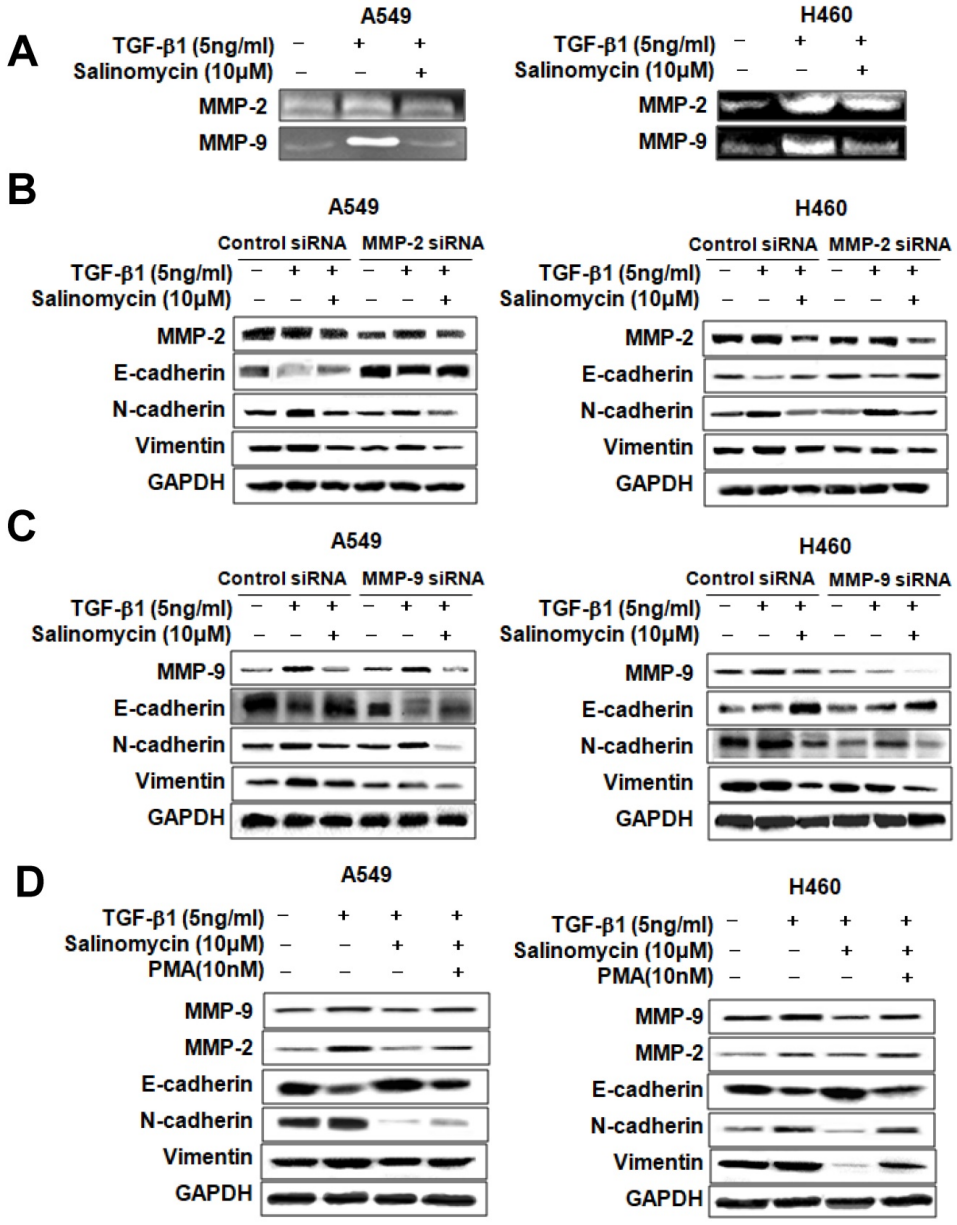
Sal's effects on TGF-β1-induced EMT through MMP-2 and MMP-9 signaling. (A) A549 and H460 cells were stimulated with 5 ng/mL TGF-β1 for 2 h and then incubated with 10 µM Sal for 24 h. The supernatants were analyzed by gelatin zymography to measure MMP-2 and MMP-9 expression. (B, C) Effects of MMP-2 or MMP-9 inhibition on TGF-β1-induced EMT by Sal. The Cells were transfected with MMP-2 or MMP-9 siRNA and then further incubated in the presence of Sal for 24 h. (D) The cells were treated with an MMP total activator, PMA, and then incubated further in the presence of Sal for 24 h. The cell lysates were routinely prepared, and alterations in EMT hallmarks were determined through Western blotting. Similar data were obtained from three independent experiments.

*Sal inhibits TGF-β1-induced EMT in NSCLC cells via attenuation of the MMP-2 and MMP-9 signaling pathway.* Sal's suppressive effect on tumor cell migration and invasion points to the prior MMP expression regulation because these proteins play an essential role in local proteolysis of EMT and cell migration 21, 22. As shown in Figure 6A, gelatin zymography showed that MMP-2 and MMP-9 activity in the TGF-β1 plus Sal-treated cells was lower than that of TGF-β1-treated cells. We knocked down MMP-2 and MMP-9 by introducing siRNAs to determine their role in TGF-β1-induced EMT of NSCLC cells. The results showed that MMP-2 siRNA treatment, combined with Sal, led to TGF-β1-induced EMT attenuation (Figure 6B). In the same way, MMP-9 siRNA treatment combined with Sal led to TGF-β1-induced EMT attenuation (Figure 6C). These findings demonstrated that MMP-2 and MMP-9 knockdowns synergistically act with Sal to inhibit TGF-β1-induced EMT.

We examined whether total MMP upregulation could confer protection against TGF-β1-induced EMT attenuation by Sal. We induced MMP expression in MMP activator PMA-treated A549 and H460 cells. As shown in Figure 6D, MMP activator PMA treatment increased TGF-β1-induced MMP-2 and MMP-9 expression levels, even with Sal. In summary, these results indicated that MMP is involved in inhibiting TGF-β1-induced EMT by Sal.

## Discussion

In the present study, we re-evaluated Sal's therapeutic potential for NSCLC treatment and clarified its molecular mechanisms. We showed that Sal inhibited TGF- β1-induced EMT and suppressed lung cancer migration and invasion, which involved the AMPK/SIRT1-mediated signaling pathway.

EMT enhances cell invasion, migration, and proliferation, by conferring certain cell traits to cancer cells. Various signals induce the EMT program of cancer cells, such as the TGF-β pathway from the nearby microenvironment 23, 24. Sal is useful for chemotherapy and is involved in the proliferation, invasion, and EMT of cancer cells. Based on previous reports, Sal induced the expression of EMT markers, such as Snail, vimentin, and Zeb-1, and decreased E-cadherin expression 25, 26. In contrast, other studies have proven that Sal reversed EMT in several cancer types 27, 28. Here, we have demonstrated that Sal reversed TGF-β1-induced EMT and inhibited NSCLC cell migration and invasion. These results suggest that Sal may exert anti-cancer effects by reversing EMT.

Next, we examined AMPK expression to elucidate the specific mechanism of Sal's inhibitory effects. AMPK reportedly participates in several processes related to cell growth, energy homeostasis, metabolic diseases, and cancer cells. Recently, several studies have shown that the AMPK pathway is involved in regulating invasion and migration by reducing MMP. Also, AMPK activation is required in EMT inhibition via regulation by EMT-related makers. Thus, AMPK signaling modulation may be an essential factor in preventing cancer development and metastasis 29-31. As shown in Figure 3-4, Sal reduced TGF-β1-induced AMPK phosphorylation. These results suggest that AMPK can influence Sal's inhibitory effect on EMT, migration, and invasive potential.

SIRT1 belongs to the class III histone deacetylase (HDAC) family, where its members are mainly located in the nucleus and play essential roles in cell proliferation, apoptosis, and senescence, as well as in inflammation and metabolism 32. Furthermore, SIRT1 promotes cancer cell survival and expansion through p53 inactivation, MYC activation, and EMT 33-35. In this study, we investigated whether SIRT1 is involved in TGF-β1-induced EMT during lung cancer cell migration and invasion, and found that its expression increased in TGF-β1-induced NSCLC cells, but Sal treatment inhibited TGF-β1-induced SIRT1 expression (Figure 3, 5).

SIRT1 and AMPK act as metabolic sensors that regulate energy metabolism based on nutritional status. They perform this role independently or cooperatively by coordinating with each other and sharing a common target molecule 36, 37. In several studies, SIRT1 induced phosphorylation and inactivation by AMPK in Thr344, thus promoting p53 acetylation and apoptosis 38. In another study, AMPK was recognized as one of the downstream SIRT1 substrates and acted as a cellular metabolic stress sensor by activation via SIRT1 phosphorylation 20. We confirmed, by upregulation or downregulation, the association between SIRT1 and AMPK. SIRT1 or AMPK downregulation reduced SIRT1 expression and AMPK phosphorylation of Sal in human NSCLC cells. Also, we confirmed that MMP-2 and MMP-9 expression increases or decreases by SIRT1 upregulation or AMPK, downregulation (Figure 3-5). The results of this study suggested that SIRT1 and AMPK interacted with the TGF- β1-induced EMT inhibition by Sal.

In conclusion, we demonstrated that Sal suppresses lung cancer cell migration and invasion by inhibiting TGF-β1-induced EMT, and that it may be partially attributed to the AMPK/SIRT1 pathway. We also found that, for MMP-2 and MMP-9, the downregulation of AMPK/SIRT1 is involved in the TGF-β1-induced EMT in A549 and H460 cells. These results suggest that the function and detailed mechanism of Sal's anti-cancer effects can help develop a new and promising treatment for lung cancer patients in the future.

## Abbreviations

Sal: Salinomycin
NSCLC: Non-small cell lung cancer
EMT: Epithelial-to-mesenchymal transition
TGF-β1: Transforming growth factor-β1
MTT: 3-(4,5-dimethyl-2-thiazolyl)-2,5-diphenyl-2H-tetrazolium bromide
SDS-PAGE: Sodium dodecyl sulfate polyacrylamide gel electrophoresis
siRNA: Small interfering RNA
HDAC: Histone deacetylase
MMP: Matrix metalloproteinases
